# Survival and Favorable Neurological Outcome Following Home Delivery at 22 Weeks of Gestation: A Case Report

**DOI:** 10.7759/cureus.110854

**Published:** 2026-06-14

**Authors:** Mitsuhiro Haga, Ayumi Oshima, Yasuhisa Nagashima, Masayo Kanai

**Affiliations:** 1 Department of Pediatrics, Saitama Medical Center, Saitama Medical University, Kawagoe, JPN; 2 Fire Department, Kawagoe District, Kawagoe, JPN

**Keywords:** extremely low birth weight infant, neonatal asphyxia, out-of-hospital birth, prehospital management, vasodilator

## Abstract

We encountered a male infant with extremely low birth weight born at 22 weeks and 0 days of gestational age in the toilet at home. He was resuscitated with chest compression and bag-valve-mask ventilation by the emergency medical service crew and was transported to our institution 50 minutes after birth. The patient was intubated and given intratracheal artificial surfactant after admission. After he recovered from the initial hypotensive phase, we maintained the blood pressure at an appropriate level using nitroglycerin to avoid cerebral overcirculation. He was discharged home at 170 days of life with no signs of intraventricular hemorrhage or periventricular leukomalacia. His overall developmental quotient at one year and seven months of corrected age was 104, and he showed no apparent developmental delay. This case demonstrates that appropriate medical management can save the lives of extremely preterm infants born outside medical institutions.

## Introduction

Unplanned, out-of-hospital birth is a major challenge for healthcare providers. According to a Japanese national survey, 891 neonates were transferred by emergency medical services due to out-of-hospital births in 2015, accounting for roughly 0.00089% (891/1,005,667) of all births that year. Although out-of-hospital birth is rare, it is associated with high morbidity and mortality [1,2]. Extreme prematurity is one of the major risk factors for neonatal death [3]. Despite improvements in survival among extremely preterm infants over recent decades [4], outcomes for those born at 22 weeks of gestation remain poor, even in in-hospital settings. A Japanese national survey reported a survival rate of 63% (183/291) among actively resuscitated infants born at 22 weeks of gestation between 2018 and 2020 [5]. Poor neurological outcomes of this population are also concerning. The Japanese national database showed that 10/75 (13%) and 32/70 (46%) of surviving patients had cerebral palsy and developmental delay, respectively, in 2008-2012 [6]. Although there has been no published data on the survival and neurodevelopmental outcomes of such a population born outside the hospital, given their extreme prematurity, the chance of favorable outcomes would be much lower. To save the lives of preterm neonates born outside medical institutions, appropriate prehospital management and sophisticated neonatal intensive care are imperative. We encountered a case of an extremely preterm infant born at 22 weeks and 0 days of gestational age at home who survived to discharge without severe neurological sequelae.

## Case presentation

The patient’s mother was a 37-year-old, gravida 2, para 1, East Asian woman. She spontaneously conceived and had regular medical check-ups at a nearby obstetric clinic. The gestational age was determined by the mother’s last menstrual period with correction for first-trimester fetal ultrasonographic measurements. No abnormalities were detected during the pregnancy. At 21 weeks and 6 days of gestation, she visited the clinic with complaints of lower abdominal pain and genital bleeding. Ultrasonography showed no cervical shortening, and uterine contractions were rare on the cardiotocogram. The obstetrician allowed her to return home. Her abdominal pain increased after returning home, and at 22 weeks and 0 days, she delivered a male infant en caul in the bathroom. The mother held the intact amniotic sac containing the newborn before it fell into the toilet bowl. When the emergency medical service arrived 10 minutes after birth, the newborn within the intact amniotic sac was placed in a basin on the bathroom floor. The paramedics ruptured the membranes and clamped the umbilical cord. The infant’s heart rate was approximately 40 beats/minute, and the muscle tone and spontaneous breathing were absent. The Apgar score was 1 at 10 minutes after birth. He was resuscitated with bag-valve-mask ventilation and chest compression for 10 minutes. He responded to the resuscitation with an increase in his heart rate to 100 beats/minute, and he cried weakly. He was wrapped in an aluminum-coated polyethylene terephthalate sheet (“rescue blanket”) to maintain his body temperature and transported to our institution with continuous bag-valve-mask ventilation. During transportation, the heart rate remained around 100 beats/minute. Percutaneous oxygen saturation could not be measured because there was no appropriate pulse oximetry sensor for infants with extremely low birth weight in the ambulance.

**Figure 1:**
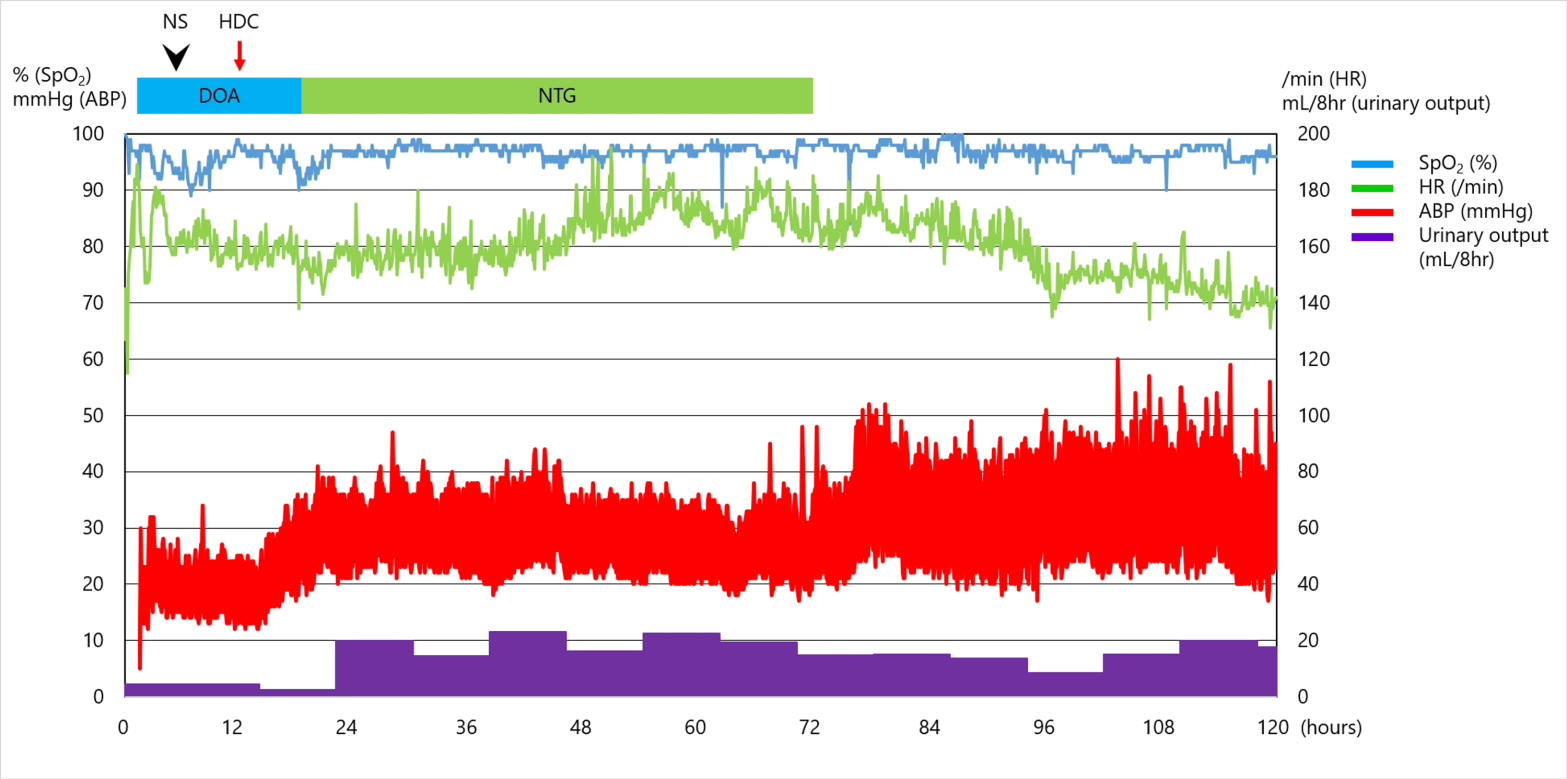
Clinical course of the patient from admission to five days of life. The patient suffered from hypotension of around 20 mmHg of the mean ABP upon admission. Dopamine infusion (8 μg/kg/minute) (light blue bar) and 10 mL/kg of normal saline administration (arrowhead) failed to increase the blood pressure. Hydrocortisone administration (2 mg/kg/dose) successfully raised the ABP. At 24 hours of life, the mean ABP rose to 30 mmHg. The echocardiograph showed mild mitral regurgitation. After starting NTG (green bar), the mean ABP stabilized around 30 mmHg, and mitral regurgitation vanished. NTG was discontinued at 72 hours of life. ABP: arterial blood pressure; DOA: dopamine; HR: heart rate; NTG: nitroglycerin; SpO_2_: percutaneous oxygen saturation

The infant was admitted to our hospital 50 minutes after birth with a weight of 492 g, a heart rate of 115 beats/minute, and a body temperature of 34.6℃. He was immediately intubated and administered artificial surfactant. The initial arterial blood gas analysis showed mild compensated metabolic acidosis; pH 7.377, partial pressure of arterial oxygen 86.7 mmHg, partial pressure of arterial carbon dioxide 29.1 mmHg, bicarbonate 17.0 mmol/L, base excess −6.8 mmol/L, and lactate 7.90 mmol/L. He suffered from hypotension, which was refractory to high-dose dopamine (8 μg/kg/minute) and a normal saline infusion (10 mL/kg) (Figure 1). Administration of hydrocortisone (2 mg/kg) successfully increased his blood pressure. Prophylactic indomethacin (0.1 mg/kg/dose) was administered after admission, and the ductus arteriosus was closed 24 hours after birth. After the closure of the ductus arteriosus, the mean blood pressure rose to 30 mmHg. Echocardiography showed mild mitral regurgitation and decreased left ventricular function due to increased afterload. We started a continuous infusion of nitroglycerin (2 μg/kg/minute) to maintain the mean arterial blood pressure at approximately 30 mmHg. After confirming improvement in cardiac function, nitroglycerin was weaned off at three days of life. On day six, he suffered from refractory hypotension requiring hydrocortisone administration. Hypotension reoccurred on day eight, and we continued hydrocortisone until day 32. He was successfully weaned from mechanical ventilation on day 63. He developed severe retinopathy of prematurity (stage 3/zone 2) in both eyes and required photocoagulation therapy. Brain magnetic resonance imaging at 42 weeks’ corrected age showed no signs of intracranial hemorrhage or periventricular leukomalacia. He was discharged home at 170 days of life (corrected age of 46 weeks and 2 days).

The patient was 2 years and 0 months of age (1 year and 7 months of corrected age) when last evaluated by us. He was walking independently and spoke a few words. His overall developmental quotient, at that corrected age, was 104 (Postural-Motor 106, Cognitive-Adaptive 105, Language-Social 100) on the Kyoto Scale of Psychological Development 2000 test. We assessed that he had no significant neurodevelopmental delays.

## Discussion

This patient’s clinical course was remarkable because he survived and achieved favorable neurological outcomes despite out-of-hospital birth at a “periviable age” of 22 weeks and 0 days of gestation. This case indicates that even though infants are born at home with severe prematurity, they have a chance of surviving without severe neurological impairment. It is likely fortunate that our patient was born en caul, which may have contributed to avoiding birth trauma and functioned as delayed cord clamping [7]. Additionally, appropriate prehospital resuscitation and treatment in an experienced neonatal intensive care unit likely also contributed to the favorable outcome.

Prehospital management is crucial for treating infants born outside healthcare facilities. The initial resuscitation with chest compression and continuous bag-valve-mask ventilation was crucial for our patient to have any chance at survival. As out-of-hospital births are rare in Japan, about half of paramedics had no experience with them in a 2015 national survey. The Japan Society of Perinatal and Neonatal Medicine promotes protocols for prehospital management of neonates through its nationwide seminars and workshops, and the neonatal resuscitation algorithm is shared among emergency medical service staff in Japan [8]. Fortunately, the three ambulance crew members had experience in transporting neonates born at home. Their experience and knowledge enabled high-quality neonatal resuscitation. Initial blood gas analysis after admission showed only mild metabolic acidosis, indicating that tissue hypoperfusion was quite limited. Appropriate temperature management is also crucial because hypothermia is associated with mortality in premature infants born outside medical institutions [1]. Although the ambient temperature on the day the patient was born was approximately 8℃, his body temperature was kept at 34.6℃ upon admission. Being wrapped in an aluminum-coated polyethylene terephthalate clearly helped preserve his body temperature. The sheet is not recommended for heat retention in preterm infants because it can only reduce heat loss from radiation and convection, but cannot prevent the loss from conduction and evaporation [9]. However, it may be considered as an option in a resource-limited situation.

Second, careful hemodynamic management after admission might also have contributed to the absence of intracranial hemorrhage in this patient to some extent. As the patient was born without receiving antenatal corticosteroids and suffered from birth asphyxia, the risk of developing germinal matrix hemorrhage (GMH) was considered high. One of the pathogeneses of GMH is the immaturity of the cerebral blood flow autoregulatory system [10]. Antenatal corticosteroids are assumed to reduce the risk of GMH by stabilizing the cerebral blood flow [11]. At our institution, we actively administer betamethasone to pregnant women from 22 weeks of gestation, in accordance with the 2021 practice advisory of the American College of Obstetrics and Gynecology [12]. As the cerebral circulation of extremely preterm infants is pressure-passive, managing adequate systemic blood pressure is crucial to prevent under- or overcirculation to the brain, especially in those not administered antenatal corticosteroids. Increased cerebral venous pressure is also a risk factor for GMH. Venous congestion can be caused by cardiac pump insufficiency; therefore, maintaining good cardiac function by reducing pre- and afterload is also imperative. Usually, blood pressure increases in a few days after birth in extremely preterm infants, and the rise in afterload can reduce the contractility of the immature heart [13,14]. We treated hypotension during the acute phase with dopamine, volume expansion, and hydrocortisone. Once the patient’s blood pressure increased, nitroglycerin infusion was effective in maintaining mean arterial blood pressure at a level that avoided decreasing cardiac function. These hemodynamic management strategies might have been effective in preventing GMH in this patient. There is no concrete evidence supporting the use of vasodilators in extremely preterm infants. Still, the number of institutions using vasodilators (nitroglycerin, milrinone, olprinone, and carperitide) for extremely preterm infants is increasing in Japan [15]. From Japan, Ichinomiya et al. reported an infant born at 21 weeks and 1 day of gestational age in the ambulance on the way to the hospital who survived to discharge [16]. However, no solid conclusion can be drawn based on these two reports. Still, Japanese management for extremely preterm infants might be capable of saving infants with severe prematurity, even if they were born outside a hospital.

## Conclusions

Extremely preterm infants who were born outside a medical institution have a chance of survival without severe neurological complications if they are given appropriate prehospital and in-hospital management. It is crucial to train emergency service members for the resuscitation of infants born outside medical institutions. Maintaining adequate blood pressure using vasodilators might prevent intracranial hemorrhage in the acute phase of extremely preterm infants. An appropriate treatment strategy for such infants born outside healthcare facilities is warranted for further consideration based on clinical experience and scientific evidence.
